# An Ecological Momentary Assessment Approach of Environmental Triggers in the Role of Daily Affect, Rumination, and Movement Patterns in Early Alcohol Use Among Healthy Adolescents: Exploratory Study

**DOI:** 10.2196/53401

**Published:** 2024-12-10

**Authors:** Maren Prignitz, Stella Guldner, Stephan Johann Lehmler, Pascal-M Aggensteiner, Frauke Nees, IMAC-Mind Consortium

**Affiliations:** 1 Institute of Cognitive and Clinical Neuroscience Central Institute of Mental Health, Medical Faculty Mannheim Heidelberg University Mannheim Germany; 2 Institute of Child and Adolescent Psychiatry and Psychotherapy Central Institute of Mental Health, Medical Faculty Mannheim University of Heidelberg Mannheim Germany; 3 Institute of Medical Psychology and Medical Sociology University Medical Center Schleswig Holstein Kiel University Kiel Germany; 4 see Acknowledgments

**Keywords:** alcohol use, adolescence, affect, rumination, ecological momentary assessment, geospatial measures

## Abstract

**Background:**

Adolescence is a period characterized by an increased susceptibility to developing risky alcohol consumption habits. This susceptibility can be influenced by social and situational factors encountered in daily life, which, in conjunction with emotions and thoughts, contribute to behavioral patterns related to alcohol use even in the early stages of alcohol experimentation, when initial experiences with alcohol are formed, and regular consumption is still evolving.

**Objective:**

This study aimed to examine the association between detailed behavioral and movement patterns, along with emotional and cognitive factors, and the early onset of alcohol use in the everyday lives of adolescents.

**Methods:**

A total of 65 healthy adolescents (33 male, twenty-nine 14-year-olds, and thirty-six 16-year-olds) underwent mobile-based ecological momentary assessments on alcohol (once a day at 9 AM, assessing alcohol use the day before), positive and negative affect, craving, rumination, and social context (6 prompts/day at 9 AM, 11 AM, 2 PM, 4 PM, 6 PM and 8 PM), type of day (weekdays or weekends, with weekend including Fridays, Saturdays, and Sundays), and using geospatial measures (specifically roaming entropy and number and type of trigger points for alcohol use met) over 14 days. After adjusting for a compliance rate of at least 50%, 52 participants (26 male and twenty-four 14-year-olds) were included in the analyses.

**Results:**

Generalized linear multilevel models revealed that higher positive affect (*b*=0.685, *P*=.007), higher rumination (*b*=0.586, *P*=.02), and a larger movement radius (roaming entropy) (*b*=8.126, *P*=.02) were positively associated with alcohol use on the same day. However, social context (*b*=–0.076, *P*=.90), negative affect (*b*=–0.077, *P*=.80), or potential trigger points (all *P*>.05) did not show significant associations. Alcohol use varied depending on the type of day, with more alcohol use on weekends (*b*=1.082, *P*<.001) and age (*t*_50_=–2.910, *P*=.005), with 16-year-olds (mean 1.61, SD 1.66) reporting more days of alcohol consumption than 14-year-olds (mean 0.548, SD 0.72).

**Conclusions:**

Our findings support previously identified factors as significant contributors to very early and low levels of alcohol consumption through fine-grained analysis of daily behaviors. These factors include positive affect, rumination, weekend days, and age. In addition, we emphasize that exploratory environmental movement behavior (roaming entropy) is also significantly associated with adolescent alcohol use, highlighting its importance as an additional factor.

## Introduction

Adolescence (spanning from ages 10 to 19 years, according to the World Health Organization [1]) represents a sensitive period characterized by rapid developmental transitions across biological, neurophysiological, psychological, and social domains [2]. These changes are important for the development of healthy behaviors in daily life [3] but can, inversely, also pose a risk for the development of unhealthy behavioral patterns. Such behaviors include the use of alcohol, which is usually initiated in midadolescence (ages 14 to 17 years) [4,5] and peaks in late adolescence around the age of 21 years [6-9]. Alcohol represents the most commonly used and abused substance during adolescence (eg, in Germany [10]) while also accounting for the most pervasive and devastating effects on life quality, general well-being, and neurodevelopmental processes [11-14]. Understanding the factors influencing very early adolescent alcohol use is crucial, as earlier initiation of alcohol consumption during this period is associated with heightened alcohol use in late adolescence and an elevated risk of developing alcohol use disorder in young adulthood [15,16]. Furthermore, alcohol use during adolescence can adversely affect structural and functional neural development [14,17], as well as various aspects of adolescent health and well-being [18,19], particularly when it involves risky behaviors such as binge drinking [13]. Therefore, elucidating the determinants of very early adolescent alcohol use, when potentially harmful patterns may be emerging for the first time, is paramount.

### A Theoretical Approach: The Incentive Sensitization Theory

Previous frameworks stem from findings on inter-individual differences in neurophysiological, genetic, personality, social, and environmental factors and their interaction in contribution to adolescent alcohol use [20-23]. The Incentive Sensitization Theory (IST) [24] proposes alcohol as a potentially rewarding stimulus that is liked at first (ie, its use is positively reinforced), for instance, due to its positive impact on mood and evolves into a so-called wanting in the course of addiction development, where its use is negatively reinforced for instance through the amelioration of negative mood states or craving [24]. Greater incentive salience is assigned to alcohol-related cues (eg, the favorite bar or the favorite alcoholic drink), which results in greater attention to these cues compared with other rewarding cues or conditions [25]. Importantly, these cues can then trigger the urge to drink alcohol [26]. Especially adolescents show hyperreactive responses to alcohol-related rewarding cues [7,27]. This model represents a theoretical approach to explaining how alcohol use transitions from early use to habitual use over time. However, specifically in the very early periods of alcohol use, that is, the onset and first experiences, information on these processes is rather scarce. Recently, through advanced methodologies, it is becoming possible to receive much more fine-grained information at the intraindividual level and in daily life to explain when and why adolescents consume alcohol.

### Daily Life Experiences Influence Alcohol Use in Adolescence

Methods to map daily life experiences, trajectories, and movements in daily life are increasingly available. These encompass ecological momentary assessments (EMAs) and GPS tracking (geospatial measures), enabling the concurrent investigation of interindividual mechanisms identified as risk factors for alcohol use (eg, IST) alongside behavioral patterns. These patterns include movements within the daily environment and exposure to specific environmental triggers (eg, nightclubs, youth gatherings, or social contexts such as being in close proximity to peers). This combination is an important addition to traditional diary methods as a resource for gaining information on individuals’ health status over time. Due to the potential to capture momentary states multiple times throughout the day, EMA methodology allows to obtain a high-resolution representation of self-reports, including emotions and thoughts as well as daily behavior and information on contexts, including social situations. Its intuitive usability further ensures greater attractiveness and, consequently, increased compliance and ecological validity compared with retrospective assessments as well as paper and pencil applications [28,29]. This also becomes significant in the realm of health research, as it provides more sensitive data regarding critical daily triggers for symptoms. Consequently, it aids in deciding on the most adaptive interventions. Previous studies using EMA have demonstrated that in adolescent and young adult alcohol users, contextual factors [30], such as the presence of peers or being in specific locations like bars or restaurants [31], along with mood-related factors, including heightened negative affect [32-34], are highly influential in initiating alcohol use. In addition, smartphone technology allows us to assess geospatial movement patterns as proxies for exploratory behaviors and environmental context factors in which specific behaviors occur (ie, alcohol-associated trigger points) and combine this with information gathered from using EMA. This provides the possibility to investigate and identify triggers with not only respect to individual feelings or thoughts but also specific contexts, including information on where (eg, private or public environments) or when (eg, time of the day, day in the week) adolescents consume alcohol [35]. These triggers can also encompass specific situational cues that are associated with alcohol use [36-38]. This information can be gained through exploratory movement patterns, which are estimated, for example, through roaming entropy, an index of “the variability in an individuals’ physical location over the course of a day” [39]. Even in the early period of alcohol use, these data could be beneficial to explore how potential trigger points develop over time. In terms of alcohol use, geospatial measures have successfully been used in app-based alcohol use disorder treatment in adults by sending alerts if patients are near alcohol-related trigger points that are risky places for lapse, relapse, or craving [40-43]. Furthermore, studies have demonstrated that neighborhood disadvantages are associated with increased substance use among adolescents [44], and adolescents tend to consume alcohol in proximity to locations where it is available for purchase [45]. However, environmental exploratory movement patterns, such as roaming entropy, have not been considered in these studies. Thus, together with EMA data, geospatial measures might allow additional insight into adolescent drinking behavior and driving factors and can add to our understanding of transitions from nonrisky to risky alcohol use, and thus inform the development of early prevention approaches, for example, in the form of ecological momentary interventions (EMI). Therefore, this study aimed to delineate the influence of daily variability in affect, craving, rumination, and presence of others (social context), together with exposure to potential environmental alcohol triggers and the characterization of environmental movement patterns on early adolescent daily alcohol use.

## Methods

### Sample

Participants were included in the study if they were either 14 or 16 years old at the time of enrollment, aiming to capture early drinking patterns typically initiated around the age of 14 years, and the period when alcohol consumption becomes more frequent and prevalent, around age 16 years [4]. This approach facilitated a better understanding and comparison of these developmental stages. In addition, participants had to be fluent German speakers, right-handed, safe to perform magnetic resonance imaging tasks (eg, no metal in the body or claustrophobia), and without any present history of psychiatric or somatic disorders [46]. Out of 72 recruited participants, 70 participants (twenty-nine 14-year-olds, thirty-six 16-year-olds, 49% female) underwent the entire assessment battery. This included a series of questionnaires (eg, sociodemographic information of participants and their parents, general alcohol consumption, stress, mindfulness, experience with meditation techniques, emotion regulation, personality, quality of life, and rumination), behavioral tasks, neuropsychological tasks in an magnetic resonance imaging scanner, and EMA. Participants received a reimbursement of €50 (approximately US $58) for their participation in the entire study. For the present purpose, only the EMA was of interest, and 65 participants underwent the entire EMA assessment.

### Instruments

#### Daily Life Experiences and Geospatial Measures

Daily life experiences were assessed by an EMA. The implementation of the EMA was done by movisensXS (movisens GmbH) [47], which allows the integration of relevant items and additional information like geospatial measures. Within the EMA assessment, participants were asked to carry along a study phone (Nokia 5 with Android version 7.1.1 Nougat) over 14 days [48]. This period was selected due to the relatively low frequency of drinking behavior observed in a healthy sample of adolescents, aiming to increase the variance in drinking occasions. A total of 6 prompts per day were presented on fixed time points (at 9 AM, 11 AM, 2 PM, 4 PM, 6 PM, and 8 PM) with 2 reminders at 5-minute intervals [48]. We thus asked participants to answer a total of 43 items regarding affect, event appraisal, craving, social context, self-esteem, and rumination within a 15-minute time window. Answering one prompt took about 2 minutes. For the current purpose, we concentrated on affect (5 items for positive affect, 5 items for negative affect, eg, “Right now I feel scared”; response scale: 1=“not at all” to 7=“very much” [49], based on Positive and Negative Affect Schedule [50]), craving (6 items, eg, “Drinking would make things seem perfect”; response scale: 1=“I totally agree” to 7=“I totally disagree,” based on ACQ [51]), social context (1 item: “Are you currently alone?” [Yes/no]) and rumination (4 items, eg, “The moment before the beep, I thought about my feelings,” reversed, response scale: 1=“I totally agree” to 7=“I totally disagree,” similar to [52]). We did not include event appraisal or self-esteem in our study. This is based on assumptions that event appraisal for good and bad experiences is reflected in positive and negative affect [53] and that fluctuations in self-esteem are rather discussed in relation to depression [54]. For positive affect, negative affect, craving, and rumination, a mean score was and for social context, a sum score was calculated for each day within each participant, with higher scores indicating higher expression of each construct (for social context, higher scores indicating more time spent alone within a day). In order to increase compliance with EMA measurements, we offered participants an additional monetary incentive (cinema voucher) if they responded to 90% or more of the EMA prompts. As compliance is central to the quality of EMA data [55], only subjects with a compliance rate of 50% or more were included in the data analyses [55], leaving a sample of 52 participants (twenty-four 14-year-olds and 26 female).

In addition to the psychometric instruments of the EMA, we gathered the GPS location of each participant on a per-minute basis, allowing us to build detailed daily movement patterns. While GPS was not linked directly to individual EMA assessments, places, or drinking events, it still enables a characterization of daily activity of and environmental influences on the participants. In addition, participants provided information on places they visited frequently during the EMA assessment (what or who was visited and where was the place or address).

#### Alcohol Use

Our main alcohol outcome was assessed within the EMA once a day, within the 9 AM prompt. Participants were asked about their alcohol use the day before (1 item: “Did you drink alcohol yesterday?” [Yes/No]). This method was used to capture every occurrence of alcohol use the day before, since the last prompt of EMA reached participants at 8 PM, and it is conceivable that participants used alcohol even after this time point [56]. If participants missed the 9 AM prompt, we coded alcohol use for the day before as missing data. In addition, as a control, we included the sum score (range 0 to 40) of the 10-item self-report Alcohol Use Disorder Identification Test (AUDIT) [57] to check whether general alcohol use is generally associated with daily alcohol patterns.

### Data Analysis

#### Geospatial Measures Preprocessing

To use geospatial measures in our model, we preprocessed GPS information. In particular, we calculated 2 measures based on individual trajectories: Roaming Entropy as a marker of environmental exploratory movement and the contact with potential alcohol trigger points, that is, locations where alcohol use might be especially salient. All analyses were done using R (R Foundation of Statistical Computing) [58].

Roaming entropy (RE) is an information-theoretic measure used to quantify the variability of a movement trajectory. Specifically, it calculates the entropy of a trajectory based on unique places visited [55]. Places are defined as distinct latitude-longitude-pairs, rounded to the fourth decimal place [39,59], providing an accuracy of approximately 11 m around the participant, which corresponds to the size of a small house or street section.

For each participant and each day separately, Roaming Entropy was calculated using the following formula:



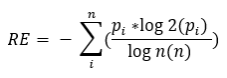



**Figure 1:**
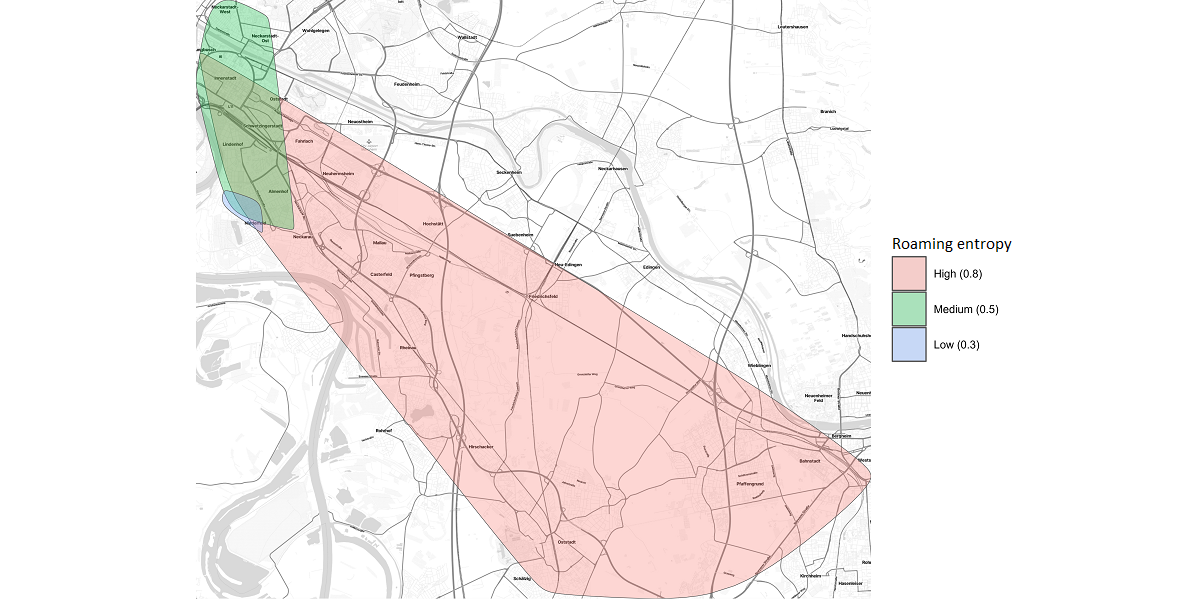
Abstract examples of 3 participants categorized by their roaming entropy within this study, depicting high (red), medium (green), and low (blue) levels. While roaming entropy primarily quantifies movement variability rather than area, it frequently aligns with larger areas covered.

Here, *p_i_* is the probability (estimated by the relative within-day frequency) that the participant is at location *i*, while *n* is the total number of unique locations visited during that day. RE can take values from zero to one, with zero meaning a participant did not move at all during the day and one meaning that every place visited was unique [39]. Figure 1 shows exemplary movement patterns with high, medium, and low RE.

Please note, the map tiles are by Stamen Design, under CC BY 4.0. Data by OpenStreetMap, under ODbL.

To calculate the contact of each participant with trigger points (TPs), we had to use additional data sources to add semantic meaning to locations. We used geolocalized information from “OpenStreetMap” (OpenStreetMap Stiftung) [60] and the “Kulturatlas Mannheim” (Stadt Mannheim) [61] to identify bars, clubs, parks, and other potential places of interest. These places were selected based on insights from previous research indicating that adolescents typically consume alcohol in places where it is legally permissible in Germany (such as private homes, friends’ homes, bars, or restaurants for 16-year-olds for specific alcoholic beverages) and that alcohol consumption among adolescents often occurs in social settings (such as clubs, meeting spots, etc). In addition, we used information provided by participants regarding their frequently visited places during the assessment period to distinguish between places of residence, study, or social interactions, such as meeting with peers.

**Figure 2:**
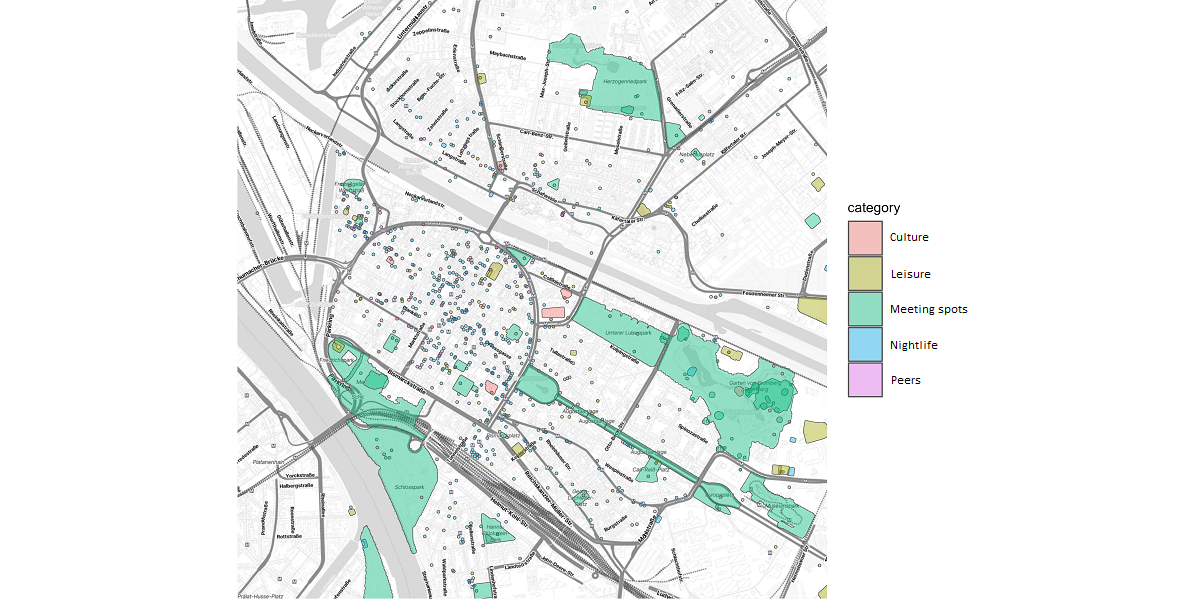
Trigger point categories within city of Mannheim, Germany.

Using these data sources, we defined categories of potential TPs. To the best of our knowledge, there is currently no standardized framework for classifying potential TPs for adolescent alcohol use based on geospatial features. Therefore, we had to create the categories by considering the available tagged locations in the data and the information provided by participants. We defined categories of TPs related to distinct social situations likely associated with alcohol use in adolescents. The five defined categories were “nightlife,” “culture,” “leisure,” “peers,” and “meeting spots,” with most of these categories standardized across the entire sample (all locations grouped under each category can be found in Table S1 in Multimedia Appendix 1). Figure 2 shows a view of the city of Mannheim, Germany, with all places categorized accordingly. Please note, the map tiles are by Stamen Design, under CC BY 4.0. Data by OpenStreetMap, under ODbL.

For each subject, we tracked how often, during one day, they would get in contact with each of the TP categories. Contact was defined by physical closeness (within 15 m) with places belonging to a category. Based only on GPS trajectories, without further detailed information on the locality and participant’s direction of view, any measurement of closeness can only be a rough estimate. In this context, the radius of 15 m was chosen as a reasonable tradeoff between accuracy and precision. Small changes in the radius did not affect our model’s results. Continuous contact (eg, many minutes or hours at the same location) was not accumulated over time but rather counted as one contact. It was possible to be in contact with multiple types of TPs at the same time.

#### Effects of Daily Life Experiences and Geospatial Information on Daily Alcohol Use

For a comprehensive examination of daily alcohol use in relation to daily life experiences and geospatial information, we used binomial generalized multilevel models with logit link function. These models are used to conduct logistic regression analyses of alcohol use as a binary outcome for each day and subject. For this purpose, the alcohol item, which was assessed on the following day, was shifted to the day before, and thus, the alcohol use of the respective day was related to the corresponding life experiences of that day. Alcohol use was predicted then by daily life experiences as independent variables assessed by EMA: positive affect (PA), negative affect (NA), craving, rumination, and social context. In addition, we used the geospatial measures TPs and RE of each day as independent variables, as well as a binary classification of assessment day into weekday (Monday, Tuesday, Wednesday, and Thursday) or day of weekend (Friday, Saturday, and Sunday) and personal level variables age and sex. To account for intraindividual correlation, we used a generalized linear multilevel model approach with a fixed slope and a random intercept for each participant, with participant as random effect and independent variables (PA, NA, craving, rumination, social context, TP, RE, weekday, age, and sex) as fixed effects.

Not all participants responded to every EMA prompt on each day. Requiring participants to answer all prompts to be included in the analyses is an open discussion in the EMA research, as it would always result in a significant reduction of the sample size [55]. Similarly, in our study, requiring participants to answer all 6 prompts would significantly reduce the sample size, making it too stringent as a cutoff criterion. Conversely, setting a cutoff too low could potentially introduce bias control challenges, particularly if nonresponse is related to drinking behavior. Therefore, we calculated a model that strikes a balance between the number of answered prompts (here, 4 prompts per day) and the number of participants, including all participants in the analysis. This approach aligns with previous work [55]. In addition, we calculated 5 additional models with varying levels of prompt completion (1 prompt/day, 2 prompts/day, 3 prompts/day, 5 prompts/day, and 6 prompts/day) to assess the stability of the results and the impact of participant compliance (more details in Figure S1 in Multimedia Appendix 1). For our main model, we evaluate the overall significance by comparing the marginal or null-model (model with only random effects) deviance to the conditional model (model containing random and fixed effects) deviance by chi-square tests. The significance of random effects is tested using the Wald test.

All analyses were done with R [58] using the lme4 package [62] and a significance threshold of *P*<.05. To check for overall effects of sex, age, and the COVID-19 pandemic restrictions, we used *t* tests for independent measures to compare the overall reported days of alcohol use within the 14-day assessment period. Since the data acquisition period coincided with the COVID-19 pandemic in Germany [63], we decided to check for these potential differences in our sample. We also evaluated the correlation of the AUDIT sum score in the sample with the number of drinking days during the EMA assessment and tested whether compliance rate had an influence on all assessed variables with *t* tests.

### Ethical Considerations

Within our subproject of the IMAC-Mind consortium [64], participants were recruited through advertisements in schools in Mannheim, Germany, through social media, and with the help of the local registration office of Mannheim. Both participants and their primary caretaker gave written informed consent before study participation. The ethics committee of Medical Faculty Mannheim, Heidelberg University, approved the study (2007-024-N-MA).

## Results

### General Sample Information

There was no significant difference in alcohol use between participants doing the EMA under COVID-19 restrictions compared with participants doing the EMA before COVID-19 occurred (t_50_=–0.372, *P*=.71), and no significant differences in alcohol use due to sex (t_50_=0.199, *P*=.85). However, there was a significant effect for age (t_50_=–2.910, *P*=.005), with 16-year-olds (mean 1.61, SD 1.66) reporting significantly more alcohol drinking days than 14-year-olds (mean 0.548, SD 0.72). We noted a significant positive correlation (*r*=0.49, 95% CI 0.26 to 0.68; *P*<.001) between reported alcohol days within the EMA and AUDIT sum score. Additionally, there was a significant sex difference in rumination (t_50_=–2.513, *P*=.02), with males having lower scores (mean 1.56, SD 0.513) than females (mean 2.08, SD 0.907) and a significant difference between participants doing the EMA under COVID-19 restrictions compared with participants doing EMA before COVID-19 occurred in negative affect (t_50_=–2.091, *P*=.04) with participants doing EMA before COVID-19 having lower scores (mean 0.89, SD 0.67) than participants doing EMA under COVID-19 restrictions (mean 1.26, SD 0.55) and social context (t_50_=2.027, *P*=.048) with participants doing EMA before COVID-19 having higher scores (mean 0.549, SD 0.215) than participants doing EMA under COVID-19 restrictions (mean 0.438, SD 0.162). All other variables were comparable between males and females and between 14- and 16-year-olds (all *P*>.05; more details in Table 1 and Table 2, and Figure S2 in Multimedia Appendix 1). Furthermore, no significant differences were observed in any of the variables between participants who did not meet the inclusion criteria of compliance (>50%) and those who did meet these criteria (all *P*>.05).

**Table 1:** Distribution of general sample characteristics within the total sample and separated by age.

Variable			Total, n		14-year-olds, n		16-year-olds, n
**Sex**							
	Male	26		12		14	
	Female	26		12		14	
**EMA** ^a^							
	Before the COVID-19 pandemic	15		7		8	
	During the COVID-19 pandemic	37		17		20	
**Current activity or job**							
	Student	50		24		26	
	Other activity or job	2		0		2	
**School type**							
	Middle school^b^	2		0		2	
	Comprehensive school^c^	3		0		3	
	Academic high school^d^	43		23		19	
	Other	2		1		2	
**Highest graduation of the father**							
	Certificate of secondary education^e^	8		3		5	
	Middle school^b^	3		3		0	
	Qualification for access to higher education^f^	24		11		13	
	University degree	16		7		9	
	Other	1		0		1	
**Highest graduation of the mother**							
	Certificate of secondary education^e^	2		0		2	
	Middle school^b^	9		5		4	
	Qualification for access to higher education^f^	28		14		14	
	University degree	12		4		8	
	Other	1		1		0	

^a^EMA: ecological momentary assessment.

^b^Corresponds to “Realschule” in the German educational system.

^c^Corresponds to “Gesamtschule” in the German educational system.

^d^Corresponds to “Gymnasium” in the German educational system.

^e^Corresponds to “Hauptschulabschluss” in the German educational system.

^f^Corresponds to “Abitur/Fachabitur” in the German educational system.

**Table 2:** General information about ecological momentary assessment for the total sample and separated by age.

	Total, mean (SD)	14-years-old, mean (SD)	16-years-old, mean (SD)
Answered prompts (max^a^ possible: 84)	65.192 (14.712)	62.7 (14.3)	67.3 (15.0)
Compliance rate	0.797 (0.140)	0.771 (0.139)	0.818 (0.140)
Positive affect	3.70 (0.803)	3.66 (0.803)	3.74 (0.816)
Negative affect	1.15 (0.607)	1.08 (0.534)	1.22 (0.666)
Craving	1.50 (0.765)	1.28 (0.495)	1.68 (0.905)
Rumination	1.82 (0.774)	1.66 (0.475)	1.96 (0.947)
Social context	0.470 (0.184)	0.493 (0.178)	0.450 (0.190)
Alcohol use days	1.115 (1.409)	0.542 (0.721)	1.61 (1.66)

^a^Max: maximum.

### Effect of Daily Life Experiences and Geospatial Measures on Daily Alcohol Use

An overview of the model statistics can be found in Table 3. The model comprises a total of 587 observation days, which is the sum of the days on which participants completed at least 4 prompts per day. There is high variability between participants, with a range of 4 days included in the model for the participant with the fewest days and 14 days for the participant with the most days (mean observation days per participant: 11.29, SD 3.15). Comparing the deviance of the conditional or full model (with fixed and random effects) to the marginal or null model (containing only fixed effects), we see an overall significant difference. The pseudo *R*² of the generalized linear mixed models for both conditional and marginal models show weak to moderate fit. While some predictors remained stable, there was variability across models with different amounts of prompts included (Table S2 in Multimedia Appendix 1 shows detailed results of models with 1, 2, 3, 5, or 6 prompts included). In the model analyzing 4 prompts per day (more details in Table 3), predictors of alcohol use were positive affect (*b*=0.685, *P*=.007), rumination (*b*=0.586, *P*=.02), weekend day (*b*=1.082, *P*<.001), and RE (*b*=8.126, *P*=.02). All other predictors did not reach significance in this model. 

**Table 3:** Detailed outcome of generalized linear mixed model for daily life experience and geospatial variables (roaming entropy and potential trigger points) on drinking behavior for at least 4 prompts per day.

Parameters					Values
**4 prompts**					
	Participants, n				52
	Total observation days^a^, n				587
**Overall model design**					
	Marginal (pseudo) *R²*				0.244
	Conditional (pseudo) *R²*				0.405
	Deviance				377.40
	Null model				332.83
	*df*				14
	Chi-square				44.571
	*P* value				<.001
**Predictor**					
	**Intercept**				
		*b*		–3.519	
		SE		1.910	
		*z* score		–1.843	
		*P* value		.07	
	**Positive affect** ^b^				
		*b*		0.685	
		SE		0.253	
		*z* score		2.703	
		*P* value		.007	
	**Negative affect**				
		*b*		–0.077	
		SE		0.303	
		*z* score		–0.252	
		*P* value		.80	
	**Craving**				
		*b*		0.348	
		SE		0.189	
		*z* score		1.844	
		*P* value		.07	
	**Rumination** ^b^				
		*b*		0.586	
		SE		0.241	
		*z* score		2.434	
		*P* value		.02	
	**Social context**				
		*b*		–0.076	
		SE		0.584	
		*z* score		–0.131	
		*P* value		.90	
	**Weekend** ^b^				
		*b*		1.082	
		SE		0.305	
		*z* score		3.534	
		*P* value		<.001	
	**Roaming entropy** ^b^				
		*b*		8.126	
		SE		3.525	
		*z* score		2.305	
		*P* value		.02	
	**Culture (trigger)**				
		*b*		0.015	
		SE		0.542	
		*z* score		0.027	
		*P* value		.98	
	**Nightlife (trigger)**				
		*b*		0.040	
		SE		0.305	
		*z* score		0.132	
		*P* value		.90	
	**Leisure (trigger)**				
		*b*		0.030	
		SE		0.383	
		*z* score		0.078	
		*P* value		.94	
	**Meeting spots (trigger)**				
		*b*		–0.081	
		SE		0.210	
		*z* score		–0.385	
		*P* value		.70	
	**Peers (trigger)**				
		*b*		–0.005	
		SE		0.460	
		*z* score		–0.010	
		*P* value		.99	
	**Age**				
		*b*		0.721	
		SE		0.457	
		*z* score		1.579	
		*P* value		.11	
	**Sex**				
		*b*		–0.295	
		SE		0.451	
		*z* score		–0.654	
		*P* value		.51	

^a^Number of days with at least 4 answered prompts per day summarized over all participants.

^b^Predictors are significant at *P*<.05 or below.

## Discussion

### Interpretation of Results

The aim of this study was to examine the influence of daily life experiences, such as affect, craving, rumination, and social context, together with the exposure to potential environmental alcohol triggers (TP) and environmental movement patterns (RE) in contributing to daily alcohol use in healthy adolescents in the very early period of alcohol experience. Our exploratory results show that daily variations in positive affect, rumination, and RE can predict drinking behavior in healthy 14- and 16-year-old adolescents. We identified core concepts that might represent a pattern associated with a higher risk for the development of risky alcohol use.

Interestingly, positive affect (not negative affect) was significantly positively associated with alcohol use on that day in our sample, with robust effects across varying levels of compliance rates (more details in Table S2 in Multimedia Appendix 1). From previous research concerning affective states on alcohol use (stress reactivity theory [5]), it would be expected that alcohol use would follow negative affect, representing a coping strategy to deal with unwanted and aversive emotions [65,66]. In contrast, our results indicate that during early alcohol use, with a low level of frequency, that is, seldom use, adolescents use alcohol when experiencing positive emotions during the day, suggesting it might be used to enhance positive emotions. This is in line with previous work on alcohol-drinking motives in adolescents [18,67]. In addition, adolescents often underestimate the risks of alcohol use and have positive attitudes toward alcohol [68]. However, it is important to note that our data does not allow for the derivation of directionality between positive affect and alcohol use. This limitation stems from the retrospective assessment of alcohol use in this study, conducted without further situational or contextual information. It is plausible that either affect may change as a result of alcohol use, or that situational features prompt both positive affect and alcohol use (eg, a celebratory event, more details in Table S3 in Multimedia Appendix 1), or that adolescents experiencing positive emotions are more likely socialize and thus consume alcohol with peers. Future studies could address this by adopting a more detailed assessment of alcohol use, including information on when, where, and in what context alcohol was consumed, or by using event-related sampling schemes to specifically collect data when alcohol is used.

Furthermore, another central factor in association with alcohol use was rumination, with higher rumination being associated with a higher probability of alcohol use on that day. This association was rather weak compared with the other predictors, and rumination ratings were generally low, but the results are still robust across differing compliance thresholds (more details in Table S2 in Multimedia Appendix 1). Thus, our data suggests that already in early, as of yet non-hazardous alcohol-using adolescents, rumination is a factor that contributes to momentary alcohol use. As we outlined above, we cannot infer directionality from our data. However, it can be speculated that even at early use, alcohol might be used to relieve negative thoughts, posing a risk factor for the development of risky drinking patterns. This is in accordance with a body of work investigating rumination as a predisposing factor of alcohol use in patients with alcohol use disorder [69] and also in adult [70] and adolescent [71,72] regular drinkers. Furthermore, our results on rumination and positive affect might be cautiously interpreted in regard to the IST [72], representing the rather prominent “liking” mechanism of early alcohol use (experiencing positive affect) [73,74] and a risk potential already at early stages for a “wanting” mechanisms (relieving rumination), which might later generalize from momentary to more stable patterns, as more habitual alcohol use develops.

Our results regarding environmental exploratory movement patterns, such as RE and potential alcohol trigger points (TPs), suggest that, unlike individuals with alcohol use disorder or heavy drinkers [31,42], adolescents in the earlier stages of alcohol use may not yet have established stable environmental cues or locations that consistently trigger alcohol use, neither on weekdays nor on weekends (more details in Table S4 in Multimedia Appendix 1). Furthermore, places potentially associated with alcohol use were visited similarly during both weekdays and weekends (more details in Figure S3 in Multimedia Appendix 1). However, it is important to consider that we used a “standardized” categorization of trigger points across the entire sample. Future studies could benefit from assessing more personalized potential trigger points for adolescents by directly asking them where they typically consume alcohol and associating these locations with EMA-based information such as affect or rumination.

Nonetheless, our results suggest that these TPs might indeed be developing, as indicated by higher RE. RE emerged as a consistently robust predictor of alcohol use across various models (more details in Table S2 in Multimedia Appendix 1). It could be speculated that this association is driven by the fact that RE may lead to increased exposure to potential alcohol contexts, such as alcohol advertisements, bars, drinking opportunities, or social contexts. However, there may be other factors contributing to this relationship. For instance, higher RE could be linked to the motivation to drink, such as prosocial drinking motives [67,75], or it could reflect a decrease in daily structure and reduced parental supervision [76,77] or a lack of scheduled activities [78-80], such as regular participation in sports clubs. In addition, it is possible that RE varies substantially within individuals from day to day due to planned behaviors related to alcohol consumption on drinking days compared to nondrinking days [81]. Exploring the relationship between RE and these factors influencing alcohol use warrants further investigation in future studies, potentially using event-related sampling schemes to align movement patterns with alcohol use patterns. In addition, in order to learn more about the development of alcohol use disorders, it would be beneficial to examine whether a similar association with alcohol use remains in adolescents with alcohol use disorders.

Finally, our results indicate that alcohol use also seems to be dependent on adolescents’ age and day of the week. These findings corroborate previous work showing alcohol use increases with age [82] and that adolescents mainly use alcohol on weekends [83,84] when there are usually no obligations to be fulfilled, but there is instead leisure time to spend with peers. Importantly, our results showing adolescents drink more on weekend days might reflect a particular behavioral pattern of adolescence that can change over time (less drinking, but also during the week) or become more pronounced (development towards alcohol addiction).

Our results have implications for prevention approaches, which should sensitize adolescents to alcohol use and its consequences and risks at an early stage before alcohol use manifests in hazardous or risky patterns. At this stage, adolescents might be less aware of the risks concerning alcohol, such as a gradual beginning that might cascade into problematic alcohol use [85]. A relevant concept of prevention might be mindfulness, which has already been successfully used in alcohol addiction treatment [86,87]. However, our results on trait attention regulation, as a relevant part of mindfulness [88], do not show a relevant influence on daily alcohol use (more details in Table S5 in Multimedia Appendix 1). It can be assumed that both mindfulness and attention regulation might be situation-dependent (eg, someone is stressed or in a hurry vs relaxed) and thus may also have a situation-dependent influence on alcohol use.

### Limitations

Several limitations of this study should be noted. First, we assessed alcohol use only once a day in the morning, inquiring about alcohol use on the previous day. Consequently, we cannot infer the directionality underlying our findings nor establish any causal relationship. In addition, we are unable to link specific alcohol use episodes to momentary ratings or motives (EMA), social contexts, event appraisal, fluctuations in self-esteem, environmental locations (geospatial information), or the amount of alcohol consumed, which would provide additional crucial information for understanding the occurrence of alcohol-related harm [89]. Future studies could benefit from using participant-initiated assessments (event-related sampling), such as when drinking episodes commence or when participants report a specific context or emotional state [31]. This would provide insights into how the appraisal of occurring events and daily fluctuations in self-esteem contribute to and interact with emotional states and environmental exploratory movement behaviors during certain alcohol consumption occasions. This, in turn, would offer a comprehensive understanding of additional factors that contribute to the initiation of alcohol use in adolescents on a daily basis and help to investigate potential comorbidities with alcohol use behaviors, such as depression, where self-esteem is discussed as a potential critical factor as well [54]. This could also help in further examining alcohol-related coping strategies and their interaction with individual factors.

Second, our study partially coincided with the COVID-19 pandemic in Germany [63], so the generalizability of our results is limited due to COVID-19 restrictions regarding environmental and social factors [90]. Nevertheless, we identify factors influencing alcohol use even under these extremely restricted circumstances. Thus, our results might be underestimated compared with times when access to alcohol and peer communities is easier.

Furthermore, we lacked information on the socioeconomic status of participants and their families. We attempted to estimate this based on the type of school attended by the adolescents (more details in Table S6 in Multimedia Appendix 1), but future studies may benefit from directly inquiring about socioeconomic status, such as family income, as this factor may also influence adolescents’ drinking behavior [91]. In addition, geospatial information like GPS shows a certain inaccuracy, especially in a dense urban environment like Mannheim, which leads to ambiguity as to what a participant is currently seeing or interacting with. This is exacerbated by the reliance on publicly available crowdsourced data sources, which correctness and relevance are not guaranteed, especially in view of temporal gaps between the EMA study and the data processing or temporal development within the assessment phase.

### Future Directions

Our results provide a first indication of how emotional and other alcohol-related factors might be related to adolescent activity profiles, that is, RE, to predict very early adolescent alcohol use. However, some of the questions raised remain unanswered. Additional EMA studies are warranted to validate the associations identified herein, including within other, more diverse (adolescent) populations, and to assess whether completing an EMA could potentially influence subsequent behavior, such as through postassessment questionnaires [92]. Once these findings are corroborated, low-threshold smartphone or app-based EMIs or ecological momentary prevention approaches could be derived from them. These interventions may assist adolescents in the early stages of alcohol use and potentially prevent the progression to risky or even abusive alcohol consumption during this vulnerable phase by bolstering resilience factors, such as psychoeducation or mindfulness [93].

Geospatial data might contribute to our understanding of developing risky alcohol use in addition to the aforementioned trigger points; in fact, internal contexts (ie, emotional trigger states) might be identified by linking emotional experience or changes in emotional states with geolocation (construction of emomaps [94]). This might be an important step for future research and would enable a link between environmental causes and behavior, such as alcohol use [95]. Building such emotional maps could be achieved by combining GPS positioning with EMA assessment as in our study, thereby enabling the exploration of the interaction of different alcohol theories with the real-life experiences of participants.

Furthermore, we observed reduced effects in certain variables when applying a stricter threshold for EMA participation, possibly due to fewer observations (more details in Table S2 in Multimedia Appendix 1). Future approaches could potentially enhance participant compliance by offering incentives, a common challenge in EMA studies [48,96], such as reducing the number of prompts per day. Our model comparison indicates that, with a fixed time-based sampling method, an optimal balance for prompts per day exists, ensuring adequate momentary data collection for statistical analysis and comparison. Study designs could be further strengthened by using a longitudinal design and a more age-diverse sample so that the effects on alcohol development could be observed over the period of adolescence.

### Conclusion

Through the use of an EMA and geospatial approach, we identified daily life positive affect, rumination, weekend days, and environmental exploratory movement behaviors, as indicated by roaming entropy, as prominent factors associated with very early adolescent drinking behavior during the initial stages of drinking pattern development. Specifically, exploring such movement patterns could serve as an important avenue for future research, offering insights into the development of trigger points from early alcohol use to potentially hazardous use or use disorders.

While environmental factors may not yet play a significant role in the early stages of alcohol use, they are recognized as critical mechanisms in later stages of alcohol use. This understanding can inform the development of future research and may be used in prevention approaches during the early stage of alcohol use.

## Appendix

Multimedia Appendix 1 An Ecological Momentary Assessment Approach of Environmental Triggers in the Role of Daily Affect, Rumination, and Movement Patterns in Early Alcohol Use Among Healthy Adolescents: Exploratory Study - Supplement.

## Abbreviations

AUDIT: Alcohol Use Disorder Identification Test
EMA: ecological momentary assessment
EMI: ecological momentary intervention
IST: Incentive Sensitization Theory
NA: negative affect
PA: positive affect
RE: roaming entropy
TP: trigger point

## Data Availability

The anonymized datasets generated during and/or analyzed during this study are available from the corresponding author on reasonable request.
