# Supplementary material for: An Ecological Momentary Assessment Approach of Environmental Triggers in the Role of Daily Affect, Rumination, and Movement Patterns in Early Alcohol Use Among Healthy Adolescents: Exploratory Study
Source: JMIR Mhealth Uhealth. 2024 Dec 10;12:e53401. doi: 10.2196/53401 (PMC11668999; doi:10.2196/53401)
Supplement: Multimedia Appendix 1 [file mhealth_v12i1e53401_app1.docx]

**An Ecological Momentary Assessment Approach of Environmental Triggers in the Role of Daily Affect, Rumination, and Movement Patterns in Early Alcohol Use Among Healthy Adolescents: Exploratory Study**

# **Supplement**

**Table S1**

Trigger point categories and related tags

| Trigger category | OSM-Tags | Kulturatlas category | Places named by participant |
| --- | --- | --- | --- |
| Culture | Amenity = Cinema | Libraries |  |
|  | Amenity = Theatre | Film |  |
|  |  | Festival |  |
|  |  | Gallery |  |
|  |  | Cabarete |  |
|  |  | Cinema |  |
|  |  | Artschool |  |
|  |  | Classic music |  |
|  |  | Dance |  |
|  |  | theatre |  |
| Nightlife | Amenity = Bar | Clubs |  |
|  | Amenity = Nightclub | Jazz |  |
|  | Amenity = Fast_food | Music event organizer |  |
|  | Amenity = Restaurant |  |  |
| Leisure | Leisure = Sports_centre | Art club | Addresses of frequently visited hobby clubs |
|  | Club = Art | Music club |  |
|  | Club = astronomy | environment |  |
|  | Club = carnival | Folk tradition |  |
|  | Club = culture |  |  |
|  | Club = environmental_protection |  |  |
|  | Club = fan |  |  |
|  | Club = game |  |  |
|  | Club = board_games |  |  |
|  | Club = music |  |  |
|  | Club = sport |  |  |
|  | Club = yes (meaning all untagged clubs) |  |  |
| Meeting Points | Leisure = Park | Parks |  |
|  | Leisure = playground | Playgrounds |  |
|  | Landuse = grass | Adventure playgrounds |  |
| Peers | Club = youth | Youthclubs | Addresses of frequently visited friends |
|  |  | Intercultural clubs |  |

*Note.* The table shows the different trigger point categories and how different tags in our three data sources were used to assign a category; OSM = OpenStreetMap.

**Figure S1**

Distribution of the total number of answered prompts for each assessment time point.


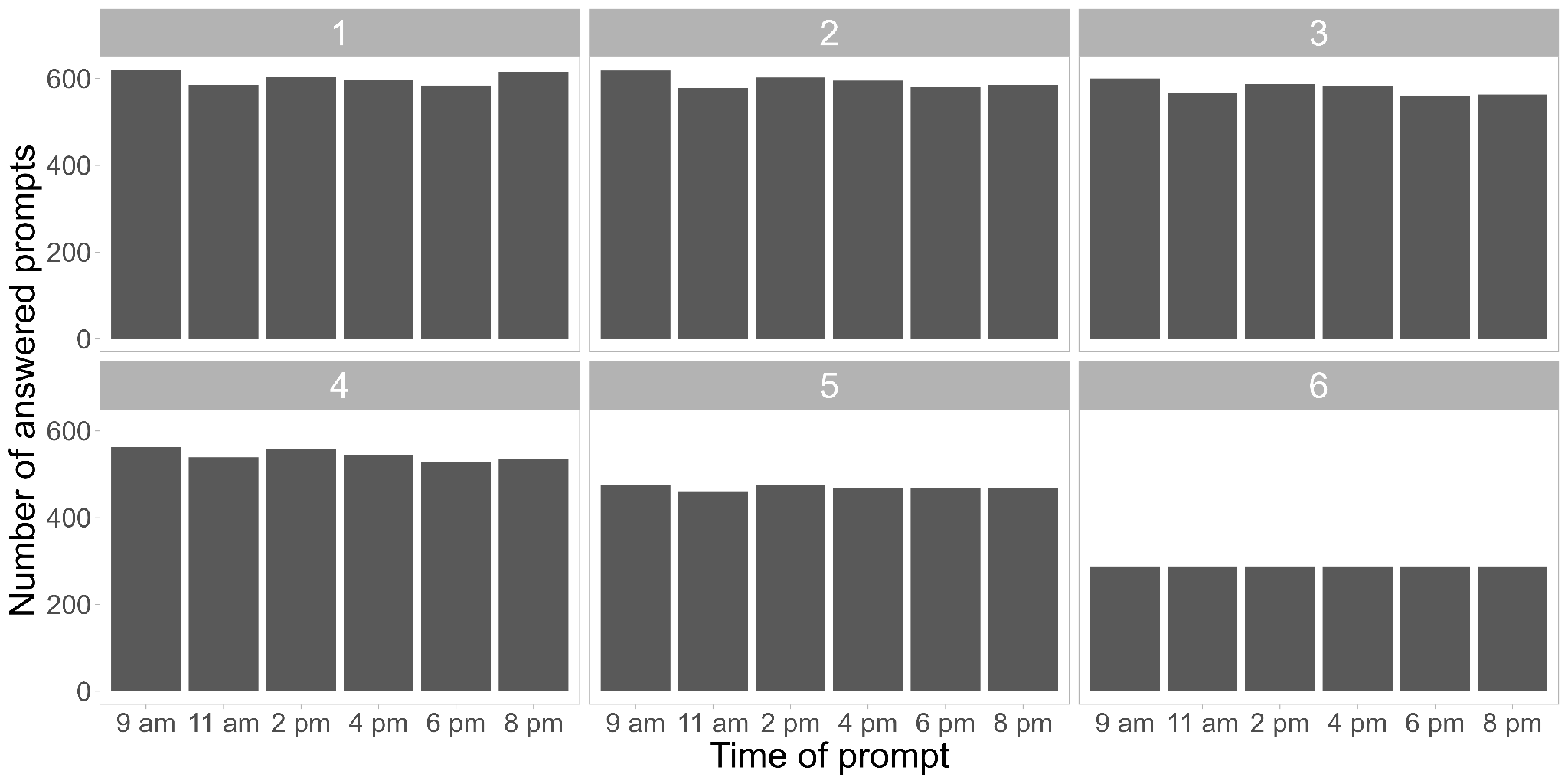


*Note.* The number of the plot refers to the models with various cut-offs: 1 = model with at least one prompt/day filled out, 2 = model with at least two prompts/day filled out, 3 = model with at least three prompts/day filled out, 4 = model with at least four prompts/day filled out, 5 = model with at least five prompts/day filled out, 6 = model with all six prompts/day filled out.

**Figure S2**

Mean value and standard errors (bars) on daily basis for three exemplary participants (marked by color) for four exemplary daily life experiences assessed by ecological momentary assessment over the course of the 14 days lasting assessment


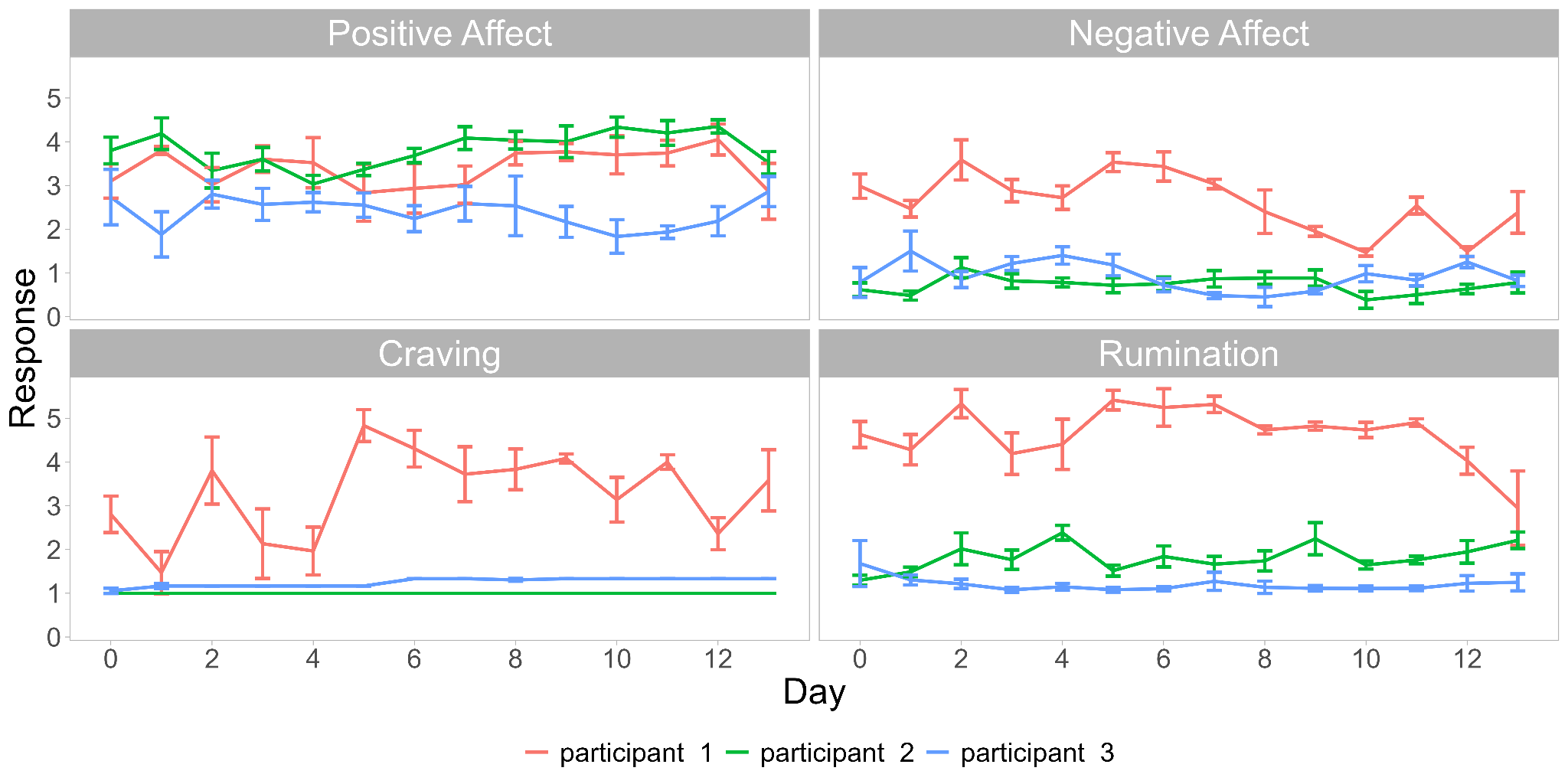


**Table S2**

Detailed outcome of GLMM models for daily life experience and geospatial variables (Roaming Entropy and potential trigger points) on drinking behavior. Models with various cut-offs for answered EMA prompts are presented within the columns

|  |  | **1 prompt** | **2 prompts** | **3 prompts** | **5 prompts** | **6 prompts** |
| --- | --- | --- | --- | --- | --- | --- |
| **N** | Subject | 52 | 52 | 52 | 51 | 48 |
|  | Total Observation Days^a^ | 731 | 689 | 647 | 478 | 271 |
| **Overall Model Sign.** | Marginal (pseudo) *R²* | 0.239 | 0.239 | 0.236 | 0.248 | 0.218 |
|  | Conditional (pseudo) *R²* | 0.382 | 0.382 | 0.378 | 0.402 | 0.404 |
|  | Deviance | 401.24 | 384.3 | 365.98 | 279.67 | 149.59 |
|  | (nullmodell) | (454.34) | (434.82) | (413.15) | (318.21) | (167.79) |
|  | *df* | 14 | 14 | 14 | 14 | 14 |
|  | *Χ²* | *53.096* | *50.562* | *47.168* | *38.538* | *18.197* |
|  | *P* value | *<.001* | *.001* | *.001* | *<.001* | *.20* |
| **Predictor** |  |  |  |  |  |  |
| *Intercept* | *b* | *-4.042* | -3.380 | *-3.511* | -3.779 | -3.693 |
|  | *SE* | 1.652 | 1.721 | *1.768* | 2.088 | 2.810 |
|  | *z* | *-2.446* | -1.964 | *-1.986* | -1.810 | -1.316 |
|  | *P* value | *.01* | .05 | *.01* | .07 | .19 |
| *Positive Affect* | *b* | *0.563* | *0.554* | *0.569* | *0.722* | 0.552 |
|  | *SE* | *0.225* | *0.229* | *0.232* | *0.275* | 0.356 |
|  | *z* | *2.500* | *2.414* | *2.454* | *2.627* | 1.550 |
|  | *P* value | *.01* | *.02* | *.01* | *.008* | .12 |
| *Negative Affect* | *b* | -0.091 | -0.005 | 0.037 | 0.026 | -0.099 |
|  | *SE* | 0.265 | 0.273 | 0.280 | 0.330 | 0.449 |
|  | *z* | -0.343 | -0.017 | 0.131 | 0.080 | -0.221 |
|  | *P* value | .73 | .99 | .90 | .94 | .83 |
| *Craving* | *b* | *0.395* | *0.36* | *0.365* | 0.329 | 0.415 |
|  | *SE* | *0.159* | *0.168* | *0.174* | 0.195 | 0.315 |
|  | *z* | *2.488* | *2.126* | *2.102* | 1.684 | 1.318 |
|  | *P* value | *.01* | *.03* | *.04* | .09 | .19 |
| *Rumination* | *b* | *0.449* | *0.538* | *0.515* | 0.561 | 0.504 |
|  | *SE* | *0.198* | *0.215* | *0.219* | 0.253 | 0.340 |
|  | *z* | *2.270* | *2.507* | *2.350* | 2.216 | 1.483 |
|  | *P* value | *.02* | *.01* | *.02* | .09 | .14 |
| *Social Context* | *b* | -0.007 | -0.087 | -0.108 | 0.058 | 0.526 |
|  | *SE* | 0.462 | 0.503 | 0.541 | 0.645 | 0.898 |
|  | *z* | -0.015 | -0.173 | -0.199 | 0.090 | 0.585 |
|  | *P* value | .99 | .86 | .84 | .93 | .56 |
| *Weekend* | *b* | *1.173* | *1.141* | *1.139* | *0.900* | 0.675 |
|  | *SE* | *0.281* | *0.287* | *0.293* | *0.331* | 0.472 |
|  | *z* | *4.174* | *3.982* | *3.889* | *2.721* | 1.431 |
|  | *P* value | *<.001* | *<.001* | *<.001* | *.007* | .15 |

**Table S2** (continued).

|  |  | **1 prompt** | **2 prompts** | **3 prompts** | **5 prompts** | **6 prompts** |
| --- | --- | --- | --- | --- | --- | --- |
| *Roaming Entropy* | *b* | *7.000* | *6.971* | *7.118* | *8.634* | 8.784 |
|  | *SE* | *3.196* | *3.249* | *3.372* | *3.835* | 5.277 |
|  | *z* | *2.190* | *2.145* | *2.111* | *2.251* | 1.665 |
|  | *P* value | *.03* | *.03* | *.04* | *.02* | .10 |
| *Culture*  *(Trigger)* | *b* | -0.050 | -0.128 | -0.046 | 0.288 | 0.562 |
|  | *SE* | 0.473 | 0.507 | 0.508 | 0.616 | 0.925 |
|  | *z* | -0.106 | -0.252 | -0.091 | 0.468 | 0.608 |
|  | *P* value | .92 | .80 | .93 | .64 | .54 |
| *Nightlife*  *(Trigger)* | *b* | 0.043 | -0.025 | -0.071 | 0.181 | -0.368 |
|  | *SE* | 0.233 | 0.261 | 0.379 | 0.357 | 0.757 |
|  | *z* | 0.186 | -0.094 | -0.052 | 0.507 | -0.487 |
|  | *P* value | .85 | .93 | .96 | .61 | .63 |
| *Leisure*  *(Trigger)* | *b* | -0.041 | -0.025 | -0.071 | 0.375 | 0.244 |
|  | *SE* | 0.363 | 0.380 | 0.379 | 0.444 | 0.586 |
|  | *z* | -0.114 | -0.01 | -0.052 | 0.845 | 0.416 |
|  | *P* value | .91 | .10 | .96 | .40 | .68 |
| *Meeting Spots*  *(Trigger)* | *b* | -0.132 | -0.133 | -0.101 | -0.204 | -0.325 |
|  | *SE* | 0.189 | 0.198 | 0.203 | 0.262 | 0.450 |
|  | *z* | -0.700 | -0.674 | -0.499 | -0.779 | -0.722 |
|  | *P* value | .48 | .50 | .62 | .44 | .47 |
| *Peers*  *(Trigger)* | *b* | -0.071 | 0.027 | -0.056 | -0.0304 | 0.240 |
|  | *SE* | 0.416 | 0.433 | 0.457 | 0.497 | 0.657 |
|  | *z* | -0.171 | 0.063 | -0.121 | -0.061 | 0.366 |
|  | *P* value | .86 | .95 | .90 | .95 | .72 |
| *Age* | *b* | *0.887* | *0.888* | 0.800 | 0.566 | 0.708 |
|  | (*SE*) | *0.413* | *0.423* | 0.425 | 0.485 | 0.662 |
|  | *z* | *2.145* | *2.100* | 1.883 | 1.167 | 1.069 |
|  | *P* value | *.03* | *.04* | .06 | .24 | .29 |
| *Sex* | *b* | -0.229 | -0.304 | -0.385 | -0.413 | -0.505 |
|  | *SE* | 0.409 | 0.417 | 0.420 | 0.478 | 0.640 |
|  | *z* | -0.559 | -0.729 | -0.917 | -0.863 | -0.789 |
|  | *P* value | .58 | .47 | .36 | .39 | .43 |

*Note.* Italic marked predictors are significant at *P* < .05 or below; *SE* = Standard error; ^a^ number of days with at least the required number of prompts per day summarized over all participants.

**Table S3**

Detailed outcome of GLMM models for at least 4 prompts/day for daily life experience and geospatial variables (Roaming Entropy and potential trigger points) on craving, rumination and positive affect.

|  |  | **Craving** | **Rumination** | **Positive Affect** |  |
| --- | --- | --- | --- | --- | --- |
| **N** | Subject | 51 | 51 | 51 |  |
|  | Total Observation Days^a^ | 574 | 574 | 574 |  |
| **Overall Model Sign.** | Marginal (pseudo) *R²* | 0.003 | 0.002 | 0.009 |  |
|  | Conditional (pseudo) *R²* | 0.752 | 0.746 | 0.746 |  |
|  | Deviance (nullmodel) | 875.91 | 865.07 | 905.06 |  |
|  | Deviance | 871.25 | 856.29 | 891.38 |  |
|  | *df* | 6 | 6 | 6 |  |
|  | *Χ²* | 4.656 | 2.782 | *13.684* |  |
|  | *P* value | 0.59 | 0.84 | *0.03* |  |
| **Predictor** |  |  |  |  |  |
| *Intercept* | *b* | *1.456* | *1.886* | *3.665* |  |
|  | *SE* | *0.119* | *0.116* | *0.120* |  |
|  | *z* | *12.221* | *16.212* | *30.685* |  |
|  | *P* value | *<.001* | *<.001* | *<.001* |  |
| *Roaming Entropy* | *b* | 0.255 | -0.428 | -0.130 |  |
|  | *SE* | 0.447 | 0.442 | 0.456 |  |
|  | *z* | 0.570 | -0.968 | -0.286 |  |
|  | *P* value | .57 | .33 | .78 |  |
| *Culture*  *(Trigger)* | *b* | 0.077 | -0.019 | 0.112 |  |
|  | *SE* | 0.086 | 0.085 | 0.088 |  |
|  | *z* | 0.891 | -0.228 | 1.272 |  |
|  | *P* value | .37 | .82 | .20 |  |
| *Nightlife*  *(Trigger)* | *b* | 0.032 | -0.013 | -0.037 |  |
|  | *SE* | 0.039 | 0.039 | 0.040 |  |
|  | *z* | 0.805 | -0.342 | -0.929 |  |
|  | *P* value | .42 | .73 | .35 |  |
| *Leisure*  *(Trigger)* | *b* | 0.013 | -0.016 | *0.166* |  |
|  | *SE* | 0.056 | 0.055 | *0.057* |  |
|  | *z* | 0.224 | -0.290 | *2.918* |  |
|  | *P* value | .82 | .77 | *.004* |  |
| *Meeting Spots*  *(Trigger)* | *b* | -0.015 | 0.006 | -0.015 |  |
|  | *SE* | 0.032 | 0.032 | 0.033 |  |
|  | *z* | -0.465 | 0.179 | -0.453 |  |
|  | *P* value | .64 | .86 | .65 |  |
| *Peers*  *(Trigger)* | *b* | 0.007 | -0.059 | 0.008 |  |
|  | *SE* | 0.059 | 0.058 | 0.060 |  |
|  | *z* | 1.220 | -1.016 | 0.127 |  |
|  | *P* value | .22 | .31 | .89 |  |

*Note.* Italic marked predictors are significant atP*p* < .05 or below; *SE* = standard error; ^a^ number of days with at least the required number of prompts per day summarized over all participants.

**Table S4**

Detailed outcome of GLMM model for at least 4 prompts/day for daily life experience and geospatial variables (Roaming Entropy and potential trigger points) on drinking behavior extended by an interaction (x) between trigger category and weekday

|  |  | 4 prompts |
| --- | --- | --- |
| **N** | Subject | 52 |
|  | Total Observation Days^a^ | 587 |
| **Overall Model Sign.** | Marginal (pseudo) *R²* | 0.243 |
|  | Conditional (pseudo) *R²* | 0.360 |
|  | Deviance (nullmodel) | 351.73 |
|  | Deviance | 310.61 |
|  | df | 19 |
|  | *Χ²* | *41.124* |
|  | *P* value | *.002* |
| **Predictor** |  |  |
| Intercept | *b* | *-7.559* |
|  | *SE* | *1.420* |
|  | *z* | *-5.325* |
|  | *P* value | *<.001* |
| Positive Affect | *b* | *0.602* |
|  | *SE* | *0.247* |
|  | *z* | *2.434* |
|  | *P* value | *.02* |
| Negative Affect | *b* | -0.148 |
|  | *SE* | 0.300 |
|  | *z* | -0.495 |
|  | *P* value | .62 |
| Craving | *b* | *0.380* |
|  | *SE* | *0.180* |
|  | *z* | *2.107* |
|  | *P* value | *.04* |
| Rumination | *b* | *0.540* |
|  | *SE* | *0.235* |
|  | *z* | *2.298* |
|  | *P* value | *.02* |
| Social Context | *b* | -0.343 |
|  | *SE* | 0.600 |
|  | *z* | -0.572 |
|  | *P* value | .57 |
| Weekend | *b* | 0.572 |
|  | *SE* | 0.361 |
|  | *z* | 1.584 |
|  | *P* value | .11 |
| Roaming Entropy | *b* | *8.929* |
|  | *SE* | *3.590* |
|  | *z* | *2.487* |
|  | *P* value | *.01* |

**Table S4** (continued).

| Culture (Trigger) | *b* | 0.131 |
| --- | --- | --- |
|  | *SE* | 0.767 |
|  | *z* | 0.170 |
|  | *P* value | .87 |
| Nightlife (Trigger) | *b* | -0.057 |
|  | *SE* | 0.611 |
|  | *z* | -0.093 |
|  | *P* value | 0.93 |
| Leisure (Trigger) | *b* | -0.108 |
|  | *SE* | 0.553 |
|  | *z* | -0.195 |
|  | *P* value | .85 |
| Meeting Spots (Trigger) | *b* | -0.502 |
|  | *SE* | 0.541 |
|  | *z* | -0.927 |
|  | *P* value | .35 |
| Peers (Trigger) | *b* | -0.263 |
|  | *SE* | 0.785 |
|  | *z* | -0.336 |
|  | *P* value | .74 |
| Culture (Trigger) x weekend | *b* | 0.361 |
|  | *SE* | 1.079 |
|  | *z* | 0.335 |
|  | *P* value | .74 |
| Nightlife (Trigger) x weekend | *b* | 0.254 |
|  | *SE* | 0.692 |
|  | *z* | 0.366 |
|  | *P* value | .71 |
| Leisure (Trigger) x weekend | *b* | -0.063 |
|  | *SE* | 0.780 |
|  | *z* | -0.081 |
|  | *P* value | .94 |
| Meeting Spots (Trigger) x weekend | *b* | 0.504 |
|  | *SE* | 0.580 |
|  | *z* | 0.870 |
|  | *P* value | .38 |
| Peers (Trigger) x weekend | *b* | 0.265 |
|  | *SE* | 1.023 |
|  | *z* | 0.259 |
|  | *P* value | .80 |
| Age | *b* | 0.663 |
|  | *SE* | 0.437 |
|  | *z* | 1.516 |
|  | *P* value | .13 |
| Sex | *b* | -0.190 |
|  | *SE* | 0.431 |
|  | *z* | -0.440 |
|  | *P* value | .66 |

*Note.* Italic marked predictors are significant at P < .05 or below; SE = Standard error; ^a^ number of days with at least four answered prompts per day summarized over all participants.

**Figure S3**

Kernel Density Estimates of the distribution of the logarithm of contacts or visits to different types of trigger points on different days of the week

**
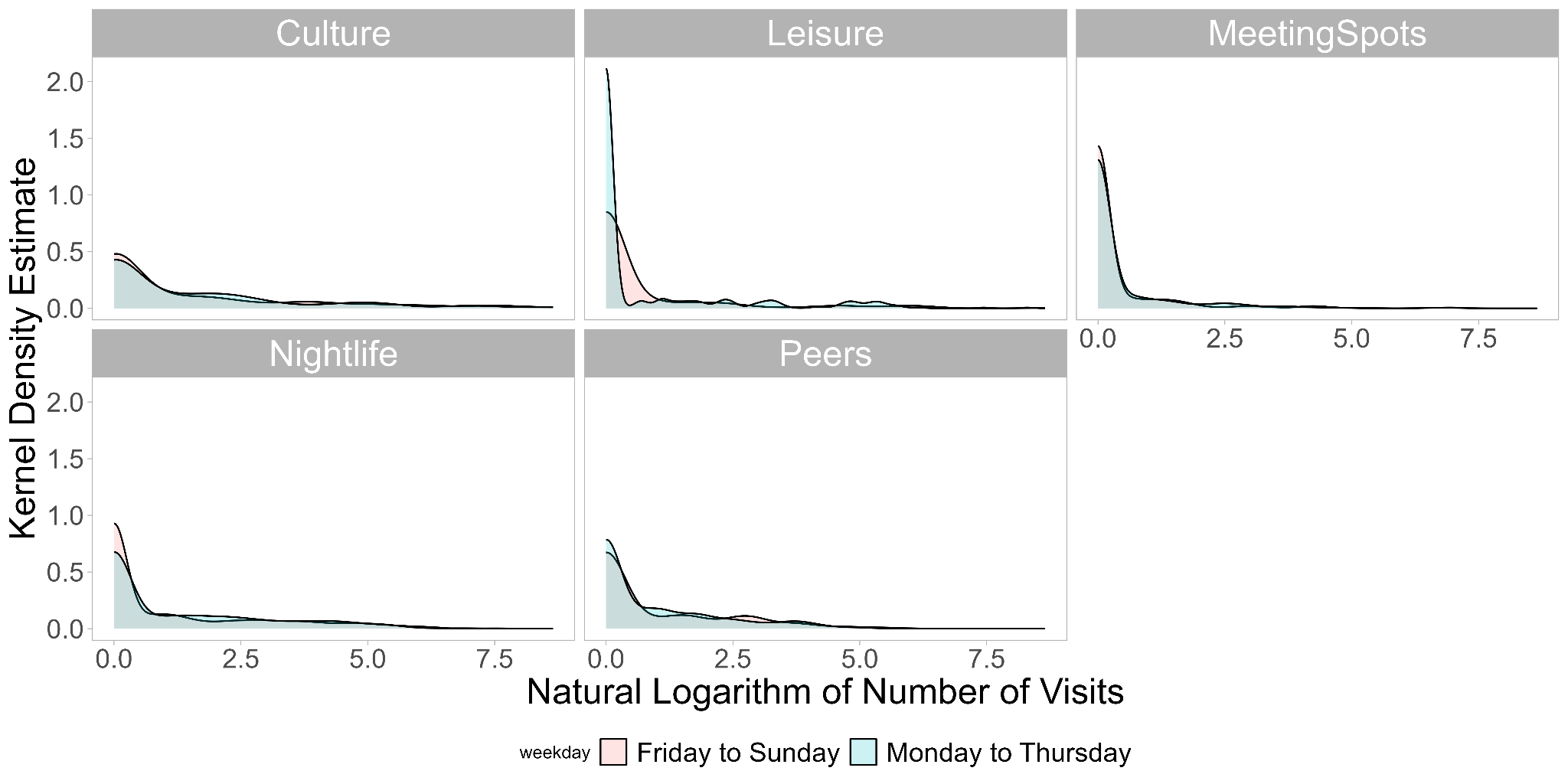
**

*Note*. Every GPS Measurement within a 50-meter buffer around the centroid of a trigger point was treated as contact.

**Table S5**

Detailed outcome of GLMM models for daily life experience and geospatial variables (Roaming Entropy and potential trigger points) on drinking behavior with additional component of trait mindful attention regulation (MAIA AR).

|  |  | **4 prompts** |
| --- | --- | --- |
| **N** | Subject | 52 |
|  | Total Observation Days^a^ | 587 |
| **Overall Model Sign.** | Marginal (pseudo) *R²* | 0.232 |
|  | Conditional (pseudo) *R²* | 0.350 |
|  | Deviance (nullmodel) | 351.73 |
|  | Deviance | 312.54 |
|  | *df* | 15 |
|  | *Χ²* | *40.115* |
|  | *P* value | *.000* |
| **Predictor** |  |  |
| *Intercept* | *b* | -4.008 |
|  | *SE* | 2.081 |
|  | *z* | -1.926 |
|  | *P* value | .05 |
| *Positive Affect* | *b* | *0.571* |
|  | *SE* | *0.254* |
|  | *z* | *2.250* |
|  | *P* value | *.03* |
| *Negative Affect* | *b* | -0.096 |
|  | *SE* | 0.301 |
|  | *z* | -0.320 |
|  | *P* value | .75 |
| *Craving* | *b* | *0.376* |
|  | *SE* | *0.180* |
|  | *z* | *2.092* |
|  | *P* value | *.04* |
| *Rumination* | *b* | *0.517* |
|  | *SE* | *0.232* |
|  | *z* | *2.230* |
|  | *P* value | *.03* |
| *Social Context* | *b* | -0.229 |
|  | *SE* | 0.592 |
|  | *z* | -0.386 |
|  | *P* value | .70 |
| *Weekend* | *b* | *0.810* |
|  | *SE* | *0.313* |
|  | *z* | *2.586* |
|  | *P* value | *.009* |

**Table S5** (continued).

| *Roaming Entropy* | *b* | *9.195* |
| --- | --- | --- |
|  | *SE* | *3.588* |
|  | *z* | *2.563* |
|  | *P* value | *.01* |
| *Culture*  *(Trigger)* | *b* | 0.267 |
|  | *SE* | 0.535 |
|  | *z* | 0.499 |
|  | *P* value | .62 |
| *Nightlife*  *(Trigger)* | *b* | 0.113 |
|  | *SE* | 0.298 |
|  | *z* | 0.381 |
|  | *P* value | .70 |
| *Leisure*  *(Trigger)* | *b* | -0.172 |
|  | *SE* | 0.399 |
|  | *z* | -0.432 |
|  | *P* value | .67 |
| *Meeting Spots*  *(Trigger)* | *b* | -0.079 |
|  | *SE* | 0.217 |
|  | *z* | -0.363 |
|  | *P* value | .72 |
| *Peers*  *(Trigger)* | *b* | -0.078 |
|  | *SE* | 0.483 |
|  | *z* | -0.162 |
|  | *P* value | .87 |
| *MAIA AR* | *b* | 0.130 |
|  | *SE* | 0.253 |
|  | *z* | 0.514 |
|  | *P* value | .61 |
| *Age* | *b* | 0.665 |
|  | *SE* | 0.436 |
|  | *z* | 1.526 |
|  | *P* value | .13 |
| *Sex* | *b* | -0.110 |
|  | *SE* | 0.466 |
|  | *z* | -0.235 |
|  | *P* value | .81 |

*Note.* Italic marked predictors are significant at *P* < .05 or below; *SE* = standard error; MAIA AR = Multidimensional Assessment of Interoceptive Awareness questionnaire (by Mehling et al., 2012), subscale Attention Regulation; ^a^ number of days with at least four answered prompts per day summarized over all participants.

**Table S6**

Detailed outcome of GLMM models for daily life experience and geospatial variables (Roaming Entropy and potential trigger points) on drinking behavior with additional component of school type.

|  |  | **4 prompts** |
| --- | --- | --- |
| **N** | Subject | 50 |
|  | Total Observation Days^a^ | 559 |
| **Overall Model Sign.** | Marginal (pseudo) *R²* | 0.265 |
|  | Conditional (pseudo) *R²* | 0.331 |
|  | Deviance (nullmodel) | 347.72 |
|  | Deviance | 302.67 |
|  | *df* | 17 |
|  | *Χ²* | *45.048* |
|  | *P* value | *<0.001* |
| **Predictor** |  |  |
| *Intercept* | *b* | -2.873 |
|  | *SE* | 2.038 |
|  | *z* | 1.410 |
|  | *P* value | .16 |
| *Positive Affect* | *b* | *0.607* |
|  | *SE* | *0.245* |
|  | *z* | *2.475* |
|  | *P* value | *.01* |
| *Negative Affect* | *b* | -0.279 |
|  | *SE* | 0.299 |
|  | *z* | -0.936 |
|  | *P* value | .35 |
| *Craving* | *b* | *0.386* |
|  | *SE* | *0.188* |
|  | *z* | *2.057* |
|  | *P* value | *.04* |
| *Rumination* | *b* | *0.632* |
|  | *SE* | *0.228* |
|  | *z* | *2.773* |
|  | *P* value | *.006* |
| *Social Context* | *b* | -0.125 |
|  | *SE* | 0.607 |
|  | *z* | -0.206 |
|  | *P* value | .84 |
| *Weekend* | *b* | *0.796* |
|  | *SE* | *0.314* |
|  | *z* | *2.536* |
|  | *P* value | *.01* |
| *Roaming Entropy* | *b* | *9.845* |
|  | *SE* | *3.600* |
|  | *z* | *2.734* |
|  | *P* value | *.006* |

**Table S6** (continued).

| *Culture* *(Trigger)* | *b* | 0.290 |
| --- | --- | --- |
|  | *SE* | 0.535 |
|  | *z* | 0.541 |
|  | *P* value | .59 |
| *Nightlife* *(Trigger)* | *b* | 0.072 |
|  | *SE* | 0.296 |
|  | *z* | 0.242 |
|  | *P* value | .81 |
| *Leisure* *(Trigger)* | *b* | -0.247 |
|  | *SE* | 0.397 |
|  | *z* | -0.623 |
|  | *P* value | .53 |
| *Meeting Spots* *(Trigger)* | *b* | -0.053 |
|  | *SE* | 0.214 |
|  | *z* | -0.246 |
|  | *P* value | .81 |
| *Peers* *(Trigger)* | *b* | -0.048 |
|  | *SE* | 0.469 |
|  | *z* | -0.102 |
|  | *P* value | .92 |
| School | *b* | 0.396 |
| (middle school) | *SE* | 1.070 |
|  | *z* | 0.370 |
|  | *P* value | .71 |
| School | *b* | 1.090 |
| (comprehensive school) | *SE* | 1.149 |
|  | *z* | 0.949 |
|  | *P* value | .34 |
| School | *b* | 0.163 |
| (academic high school) | *SE* | 0.838 |
|  | *z* | 0.194 |
|  | *P* value | .85 |
| *Age* | *b* | 0.724 |
|  | *SE* | 0.438 |
|  | *z* | 1.654 |
|  | *P* value | .10 |
| *Sex* | *b* | -0.566 |
|  | *SE* | 0.435 |
|  | *z* | -1.300 |
|  | *P* value | .19 |

*Note.* Italic marked predictors are significant at *P* < .05 or below; *SE* = standard error; School = school type coded as factor with “other” as reference class; ^a^ number of days with at least four answered prompts per day summarized over all participants.
